# Searching for radiologic and histologic evidence on live vaginal tissue: Does the G-spot exist?

**DOI:** 10.4274/tjod.galenos.2021.31697

**Published:** 2021-03-12

**Authors:** Ahmet Akın Sivaslıoğlu, Sezen Köseoğlu, Funda Dinç Elibol, Yelda Dere, Ayavar Cem Keçe, Eray Çalışkan

**Affiliations:** 1 Muğla Sıtkı Koçman University Faculty of Medicine, Deparment of Obstetrics and Gynecology, Muğla, Turkey; 2 Associate Professor, Private, Muğla, Turkey; 3 Muğla Sıtkı Koçman University Faculty of Medicine, Deparment of Radiology, Muğla, Turkey; 4 Muğla Sıtkı Koçman University Faculty of Medicine, Deparment of Pathology, Muğla, Turkey; 5 Specialist, Psychotherapist, Private, Ankara, Turkey; 6 Okan University Faculty of Medicine, Department of Obstetrics and Gynecology, İstanbul, Turkey

**Keywords:** G-spot, hyperintense focus, MRI, neurovascular tissue

## Abstract

**Objective:**

There is a growing debate on the existence of the G-spot. G-spot amplification by various surgical interventions has become mainstream for esthetic vaginal surgery despite a lack of conclusive proof of the G-spot. The aim of this study was to search for histologic evidence in regions of so-called hyperintense focus (HF) (considered as the G-spot) using magnetic resonance imaging (MRI) mapping and biopsied tissues.

**Materials and Methods:**

Fifteen patients who had grade 2 or higher anterior compartment defects were enrolled in the study. All patients were subjected to MRI. When a HF was seen, its localization, dimensions, and distances to adjacent structures were measured in images. Dissections in the anterior vaginal wall were performed in accordance with the measurements derived from MRI and tissue measuring 0.5x0.5 cm was biopsied from the determined HF.

**Results:**

An HF was determined in MRI of three (20%) patients. However, no significant neurovascular tissue density was observed histologically in any of the biopsy specimens obtained from the surgical dissections under the guidance of MRI mapping.

**Conclusion:**

Our findings denote that there is no G-spot in the anterior vaginal wall.

**PRECIS:** The G-spot does not exist.

## Introduction

The existence of the G-spot is a debatable issue in sexual medicine. Despite a lack of definitive evidence for its existence, use of the term “G-spot” has become widely accepted both in the lay media and scientific research. Moreover, although the G-spot has not been definitely shown, G-spot amplification by various surgical interventions has become mainstream for esthetic vaginal surgery.

In their observational magnetic resonance imaging (MRI) study, Maratos et al.^(1)^ claimed that the G-spot had been visualized as a hyperintense focus (HF). Hence, the main aim of this study was to shed light on this controversial issue using MRI mapping (MRIM) and to search for histologic evidence in tissues biopsied from the projection of HF.

## Materials and Methods

The study is a prospective observational study. The ethics committee of the university approved the study (decision date and number: June 18^th^, 2020-06/V). Fifteen patients who had anterior vaginal compartment defects (Ba point ^>^2 according to POP-Q) and were willing to undergo surgery between July 1^st^, 2020, and October 1^st^, 2020, were enrolled in the study. All patients were asked if they had any knowledge concerning the G-spot and whether they believed in its existence. All surfaces of the anterior vaginal wall were tactilely stimulated by starting at the urethrovesical junction and staying within the boundaries of the lateral fornix towards the anterior fornix, by making a beckoning gesture with the right-hand forefinger while wearing a sterile glove during a gynecologic examination in the lithotomy position. The patients were asked whether they had any increased sensitivity in any area during this examination.

Patients with the following were not included in the study: previous vaginal surgery, presence of concomitant apical prolapse and or paravaginal defect, history of estrogen and/or antidepressant use, postmenopausal status, a known malignancy and the patients whose coital frequency is <1/week. Informed consent regarding the MRI and surgery (biopsy + anterior compartment surgery) was given by the enrolled patients.

All patients were subjected to MRI with a 5-mm slice thickness. When a HF (putative G-spot) was seen, its localization, dimensions, distance to the hymenal ring (vaginal introitus), to the external urethral meatus and the depth from the vaginal lumen were measured on images so that the localization of the ‘‘putative G-spot’’ seen in the MRI was precisely determined and this procedure was named as “mapping of the anterior wall of the vagina.” Subsequently, each patient was enlisted for anterior compartment defect surgery.

Before the main surgery, the HF was projected on the anterior vaginal wall in accordance with the measurements derived from MRI (under the strict guidance of the mapping of the anterior wall of the vagina) and a spot was marked with a sterile pen. A tissue measuring 0.5x0.5 cm was biopsied from this region (surgical pictures, Picture 1-3). This is a novel idea and we called it MRIM.

### Statistical Analysis

Statistical analyses were performed using the Statistical Package for the Social Sciences software, version 23 (SPSS, Inc., Chicago, IL). The data are expressed as the mean and range for continuous variables, and binary variables are reported as numbers and percentages.

### Radiological Technique and Evaluation

All patients underwent pelvic MRI in the supine position using a 3T MR (Siemens Magnetom Skyra, Erlangen, Germany) before surgery. T1-weighted (W) images were obtained in axial and sagittal planes. T2-W images were acquired in axial, sagittal and transverse planes. The slice thickness of the sequences was 5 mm.

T2-W axial and sagittal images were scrutinized for the detection of a HF. If there was an HF in the images (Radiologic images, Image 5), it was recorded as a “putative G-spot” as described in the study of Maratos et al.^(1)^. The location of the HF (right or left side of the vagina), the distance between HF and vaginal introitus, the distance between the HF and the external urethral meatus, and the depth of HF’s location with respect to the vaginal lumen were measured in appropriate planes (Radiologic images, Image 1, 2). In addition, the antero-posterior diameter and area of the HF were measured in axial T2-W planes (Radiologic images, Image 3, 4). The lower abdominal MRIs were interpreted by the same radiologist.

### Anatomic Dissection

Patients underwent surgery in the lithotomy position. The surgical field was cleaned with 4% chlorhexidine and draped. The projection of HF was marked on the vaginal wall using a sterile pen according to the MRI mapping (Surgical pictures, Picture 1, 2). The vaginal mapping taken in the supine position sufficiently corresponded to the biopsies performed in the lithotomy position. Afterwards, surgical dissection was started. A full-thickness linear incision was performed starting from the urethrovesical junction and extending to the cervico-vesical junction. The bladder was dissected off the pubocervicovaginal fascia. A biopsy of 0.5x0.5 cm was taken from the projection spot of the region marked at the beginning of the procedure and was placed into 10% formaldehyde solution and sent to the pathology laboratory (Surgical pictures, Picture 3). Biopsies were taken from the pubocervicovaginal fascia (e.g. vaginal adventitia) underneath the bladder. The marking, biopsy, and anterior compartment surgery were performed by the same surgeon.

### Histologic Evaluation

The slides were ready for evaluation after routine automated tissue processing, paraffin embedding, and hematoxylin&eosin (H&E) staining. In addition, for detailed microscopic evaluation, immunohistochemistry was performed on biopsied tissues. S100 and CD34 immunostaining were used to identify neuronal and vascular structures, respectively. Three-four-mm-thick sections were cut from the paraffin blocks and immunostaining was performed automatically using a Leica Bond-Max with anti-S100 and anti-CD34 antibodies (Leica). The H&E and immunostained slides were examined for the presence and intensity of neural and vascular structures under a Nikon Ni-U light microscope (Histological images 1-6). The presence of neural bundles was verified using S100 immunohistochemistry and S100-stained neural structures were counted under the light microscope. The total count of neural structures were divided by the total microscopic area to calculate the number of neural bundles per mm^2^.

The biopsy specimens were evaluated by the same pathologist.

## Results

A total number of 15 patients were included in the study. The demographic data of the patients are given in Table 1. The mean age of the patients was 45±5.12 years.

Eleven of the 15 patients (73%) knew of the G-spot, and 4/15 (27%) did not. Interestingly, these 4 patients had heard about the G-spot, but they had no clear idea regarding its existence.

On the other hand, only 1 patient (0.06%) answered positively when asked whether she had increased sensitivity during the gynecologic examination of the anterior vaginal wall (case number 6); however, this woman had no structure compatibility (HF) with the G-spot complex in the lower abdominal MRI.

Three of 15 patients (20%) had putative G-spots (HFs) in the lower abdominal MRI (case number 4, case number 10, and case number 13). The data related to putative G-spots are given in Table 2.

All putative G-spots were detected on the left side of the vagina. The mean distance to the external urethral meatus was calculated as 38.53±6.74 (range 31-44) mm. Neurovascular tissue density was not observed histologically in any of the biopsied tissue mapped using MRI. Histologic examination of tissue samples showed only a few neural structures both in the sections stained with H&E and S100 (Histopathologic images 1-6).

## Discussion

This is the first study on live tissues searching for the G-spot both histologically and radiologically. The G-spot is defined as “a sensitive area inside a woman’s vagina that is thought to give great sexual pleasure when touched”^(2)^. Hence, it would be prudent to scrutinize the anatomy of the anterior vaginal wall. The vagina is essentially a tube that connects the uterus to the perineum. The vagina is composed of four histologic layers (internal to external): (1) Stratified non-keratinized squamous epithelium - this layer provides protection and is lubricated by cervical mucus, the vagina itself does not contain any glands, besides, this layer has no nerve fibers. (2) Elastic lamina propria - a dense connective tissue layer that projects papillae into the overlying epithelium. The larger thin-walled veins and nerve fibers are located here. (3) Fibromuscular layer - comprising two layers of smooth muscle (an inner circular and an outer longitudinal layer) and some nerve fibers. (4) Adventitia - a fibrous layer, which provides additional strength to the vagina. This layer also binds the vagina to surrounding structures.

The lower part of the vagina is innervated by the pudendal nerve, and the upper part is mainly innervated by hypogastric plexuses and splanchnic nerves. The nerve fibers of the vagina are mostly parasympathetic and arise vasodilatory effects on the erectile tissue of the vestibular bulbs and clitoris. The distal third of the vaginal wall possibly has a richer innervation and blood supply compared with the proximal third. The distal anterior vaginal wall is a highly sensitive area.

In 1950, Ernst Gräfenberg described an area along the anterior vaginal wall, close to the bladder, and noted it to be sensitive to stimulation^(3)^. Addiego described an area approximately 1.5-2 cm anterior to the urethra, associated with pleasurable sensation and enlargement by 50% during stimulation in a multiparous female patient^(4)^.

Although the first definition of the G-spot was made in the 1950s, scientific studies about its existence started to appear in the literature after the 2000s. First, in 2012, Ostrezenski defined the G-spot as fibroconnective erectile tissue on the dorsal perineal membrane in an 83-year-old fresh cadaver, approximately 16.5 mm away from the urethral meatus^(5)^. Later, in the dissection study of Ostrezenski in 2014, the author stated that a macroscopically grape-like structure in the anterior wall of the vagina was rich in neurovascular tissue and had its own ganglionic nerve^(6)^. Ostrezenki stated that 72% of G-spots were located on the left and the distance from the urethral meatus was 55 mm^(7)^. In our study, we also located hyperintense foci (e.g corresponding to the putative G-spot) and the distance from the urethral meatus was measured as an average of 39.43 mm in MRI but we could not prove its existence histologically (neural and vascular components were not observed in either H&E or S100 staining).

Some authors reacted to Ostrezenski’s assertive study about the presence of the G-spot. Hoag found that there was no microscopic structure other than the urethra and vaginal wall epithelium in the location of the putative G-spot in a study on 13 fresh cadavers with an age range of 32 to 97 years^(8)^. Hoag also emphasized that the lateral vaginal veins observed in anatomic dissection were not erectile tissue and the veins were responsible for the venous flow of the urethra, vaginal wall, and clitoris with dense vascular structure^(9)^. Our findings are in accordance with Hoag’s findings. Moreover, Puppo revised the female sexual anatomic terminology and noted that there was no G-spot and this may be scientific fraud^(10)^.

As the world became more open to sexuality in recent years, female sexuality has taken its place in the centre of sexual medicine. In this context, claiming the G-spot as a hypererogenic erectile area and amplification procedures (such as G-shot, hyaluronic acid injections, autologous adipose tissue injections) started to generate great marketing and interest. It is noteworthy that the presence of the G-spot is contradictory and the scientific background is weak regarding the benefits of amplification procedures applied to this spot. In addition, amplification interventions to the G-spot are even said to be female genital mutilation type 4. The belief that the presence of the G-spot both creates motivation for women to achieve sexual satisfaction and opportunities for those who benefit from this market.

Although the presence of a hypererogenic region in the anterior vaginal wall has been claimed to be the G-spot^(1)^, the histologic structure of the anterior wall should not be forgotten. The anterior wall of the vagina is thinner and richer in neural tissue than the posterior wall of the vagina^(11)^. Keeping in mind this anatomic information, the amplification procedures performed to the region defined as the “G-spot” in the anterior wall of the vagina may in fact cause ballooning of the anterior wall of the vagina so that penile contact of this region results in increased sexual pleasure.

In our study, only one patient (0.06%, case number 6) reported a hypererogenous region in the anterior wall of the vagina; however, we could not find a HF in the MRI of that patient.

In this study, no neurovascular element was found microscopically in biopsy specimens taken from the so-called HF (putative G spot). In the literature, cadaveric dissection studies related to the existence of the G-spot have been performed^(5,6,7,9)^. However, this study is more advanced because imaging and histologic examination were performed sequentially in live tissues in the search for the G-spot.

## Conclusion

Previous studies were performed on cadavers of elderly women, whereas ours is the first to be performed in both a younger (premenopausal) population and in living tissue. So-called HFs were seen in three patients but we could not identify any neuronal element in the biopsied tissues of these women. Our findings denote that there is no G-spot in the anterior vaginal wall. However, more imaging and histologic studies are needed to form a solid conclusion.

## Figures and Tables

**Table 1:**
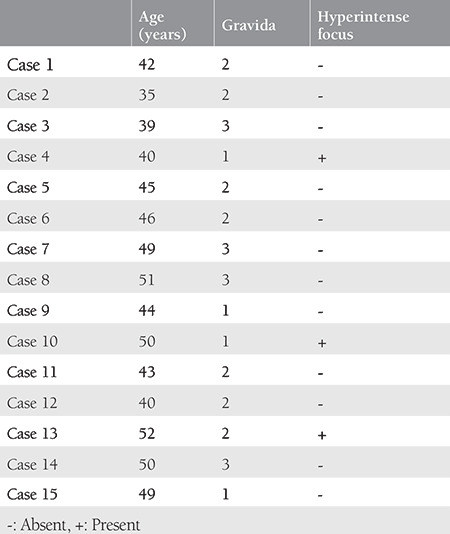
Demographic features of the patients

**Table 2:**
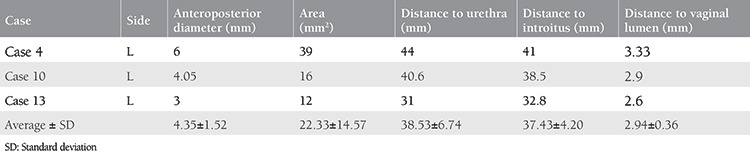
Data related to hyperintense focus (putative G-spot)

**Image 1, 2, 3, 4, 5:**
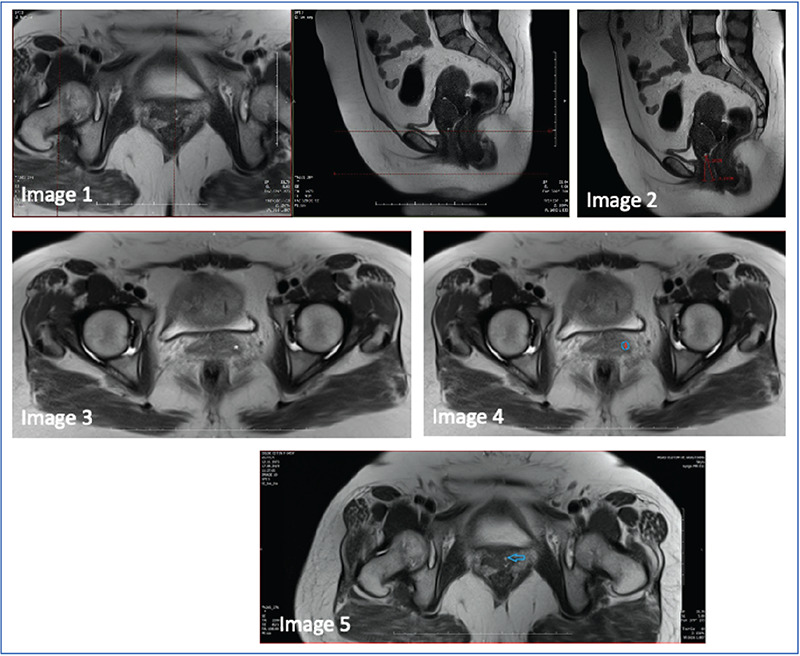
**Image 1.** Localization of hyperintense focus on axial and sagittal T2-weighted images of case number 13 **Image 2.** On sagittal T2-weighted image hyperintense focus and the distance between urethra-focus (first red line) and introitus-focus (second red line) of case 13 **Image 3.** Hyperintense focus on axial T2-weighted images marked with asterix the of case number 4 **Image 4.** Anteroposterior distance (red line) and area (blue line) of hyperintense focus on axial T2-weighted images of case number 4 **Image 5.** Hyperintense focus on axial T2-weighted image shown with blue arrow

**Image 1, 2, 3, 4, 5, 6:**
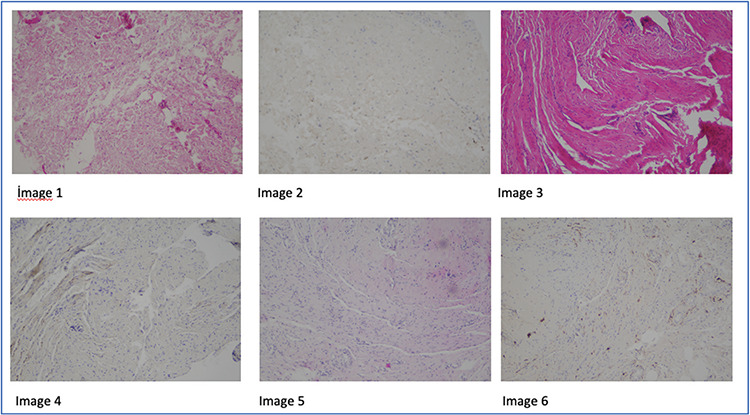
**Image 1.** Vascular lumina between the fascial bundles without neural structures, H&E x10, case number 4 **Image 2.** S100 (+) neural plexus between fascial bundles, DAB, x10, case number 4 **Image 3.** Vascular lumina in between fascial bundles without neural structures, H&E x10, case number 10 **Image 4.** No S100 (+) neural structures seen between fascial bundles DAB, x10, case number 10 **Image 5.** Vascular lumina between fascial bundles without neural structures, H&E x10, case number 13 **Image 6.** No S100 (+) neural structures between fascial bundles, DAB, x10, case 13 H&E: Hematoxylin&eosin

**Picture 1, 2, 3:**
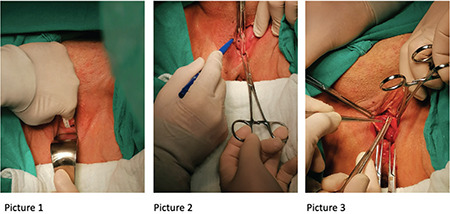
**Picture 1, 2.** The hyperintense focus marked on the vaginal wall using MRI mapping and a full-thickness linear incision from the urethrovesical junction and extending the cervico-vesical junction **Picture 3.** Taken of a biopsy of 0.5x0.5 cm from the region marked by MRIM
